# Pupil dilation signals uncertainty and surprise in a learning gambling task

**DOI:** 10.3389/fnbeh.2013.00218

**Published:** 2014-01-03

**Authors:** Claudio Lavín, René San Martín, Eduardo Rosales Jubal

**Affiliations:** ^1^Centre for the Study of Argumentation and Reasoning, Faculty of Psychology, Universidad Diego PortalesSantiago, Chile; ^2^Laboratory of Cognitive and Social Neuroscience (LaNCyS), UDP-INECO Foundation Core on Neuroscience (UIFCoN), Universidad Diego PortalesSantiago, Chile; ^3^Center for Cognitive Neuroscience, Department of Psychology and Neuroscience, Duke UniversityDurham, NC, USA; ^4^Centro de Neuroeconomía, Facultad de Economía y Empresa, Universidad Diego PortalesSantiago, Chile; ^5^Department of Neurophysiology, Max Planck Institute for Brain ResearchFrankfurt am Main, Germany; ^6^Ernst-Strüngmann Institute (ESI) for Neuroscience, Cooperation with the Max Planck SocietyFrankfurt am Main, Germany; ^7^Focus Program Translational Neurosciences (FTN), AG Molecular Imaging and Optogenetics, Institute for Microscopic Anatomy and Neurobiology, Johannes Gutenberg-University MainzMainz, Germany

**Keywords:** pupil dilation, noradrenaline, uncertainty, surprise, gambling task, learning, Iowa Gambling Task

## Abstract

Pupil dilation under constant illumination is a physiological marker where modulation is related to several cognitive functions involved in daily decision making. There is evidence for a role of pupil dilation change during decision-making tasks associated with uncertainty, reward-prediction errors and surprise. However, while some work suggests that pupil dilation is mainly modulated by reward predictions, others point out that this marker is related to uncertainty signaling and surprise. Supporting the latter hypothesis, the neural substrate of this marker is related to noradrenaline (NA) activity which has been also related to uncertainty signaling. In this work we aimed to test whether pupil dilation is a marker for uncertainty and surprise in a learning task. We recorded pupil dilation responses in 10 participants performing the Iowa Gambling Task (IGT), a decision-making task that requires learning and constant monitoring of outcomes’ feedback, which are important variables within the traditional study of human decision making. Results showed that pupil dilation changes were modulated by learned uncertainty and surprise regardless of feedback magnitudes. Interestingly, greater pupil dilation changes were found during positive feedback (PF) presentation when there was lower uncertainty about a future negative feedback (NF); and by surprise during NF presentation. These results support the hypothesis that pupil dilation is a marker of learned uncertainty, and may be used as a marker of NA activity facing unfamiliar situations in humans.

## Introduction

Deciding is a daily activity that involves a series of complex cognitive processes. The complexities are related to the lack of information and unknown environments that people usually face when making decisions. In order to understand the substrates involved in such situations, pupil dilation has been studied as a relevant marker of cognitive efforts and attention responses within different experimental contexts (Beatty, 1982; Backs and Walrath, 1992; Granholm et al., 1996). Recent studies have specified the role of pupil dilation within decision-making environments, associating this marker to variables such as uncertainty, outcome probability, surprise and valence (Satterthwaite et al., 2007; Preuschoff et al., 2011).

Pupil dilation has been linked to Locus Coeruleus (LC)—Noradrenaline (NA) activity (Aston-Jones and Cohen, 2005; Einhauser et al., 2008; Jepma and Nieuwenhuis, 2011; Kuipers and Thierry, 2011) which in turn has been related to uncertainty signaling (Rajkowski et al., 1994; Yu and Dayan, 2005). However, the precise variables that modulate pupil dilation and the way of measuring it remain controversial. While some evidence indicates that there is greater pupil dilation facing uncertainty, violation of expectations and negative outcome feedback (Satterthwaite et al., 2007), others using different tasks point out that pupil dilation changes are greater facing less uncertainty regardless of outcome valence (Preuschoff et al., 2011). This second interpretation is in line with the mentioned role of NA in uncertainty signaling, and suggests that as dopamine plays an important role in the reward pathway (Holroyd and Coles, 2002), NA is involved in a less studied circuit of uncertainty signaling involved in the learning processes of decision making in unknown environments (Nieuwenhuis et al., 2005; Yu and Dayan, 2005; Preuschoff et al., 2011; Payzan-Lenestour et al., 2013). In order to test this hypothesis, we studied pupil dilation responses in the Iowa Gambling Task (IGT; Bechara et al., 1994), a decision-making task that requires active learning from participants.

The IGT is a well-studied paradigm in which participants face a gambling situation. In each trial subjects have to choose a card from one of the four decks presented (named A, B, C and D respectively). Participants are told that a gain will always be associated with the election of the card, but that sometimes there could be a loss altogether with the gain. Unknown to participants is that two decks are more beneficial than the others. This task requires the involvement of high cognitive functions such as outcome monitoring processes, error detection, and hypothesis testing, important variables within the traditional study of human decision making (Carter et al., 1998; Gehring and Willoughby, 2002; Hewig et al., 2007).

In the IGT the underlying probability distribution associated with the decks is never presented to participants. Previous pupil-dilation paradigms provided explicit information about the probability levels of receiving either positive or negative feedback (NF), for example when participants had to bet whether an unknown card had a higher value than a known one (Satterthwaite et al., 2007), or did not let participants learn from the task (Preuschoff et al., 2011). In these cases, participants did not have to learn the probabilities associated with a given scenario, but instead they were provided during or after the decision epoch. In the IGT such information is not provided, letting subjects interact with the task in a more ecological manner. In the IGT subjects have to learn from experience, allowing a proper measure of learned uncertainty, expectations and feedback monitoring. Thus, observing pupil dilation responses toward these variables in the IGT can clarify the role of this marker and explore the hypothesis that pupil dilation signals uncertainty as a part of the LC-NA complex involved in learning within decision-making tasks.

## Materials and Methods

### Participants

Ten healthy participants with normal or corrected to normal vision performed an adaptation of the IGT (Bechara et al., 1994). Their age range was between 20 and 26 (*M* = 23.4, *SD* = 2.4; 5 women). Subjects were asked to fill and sign an informed consent before the experiment. All procedures were made in accord to the Declaration of Helsinki (World Medical Association, 2000).

### Display and Pupillometry

**Figure 1:**
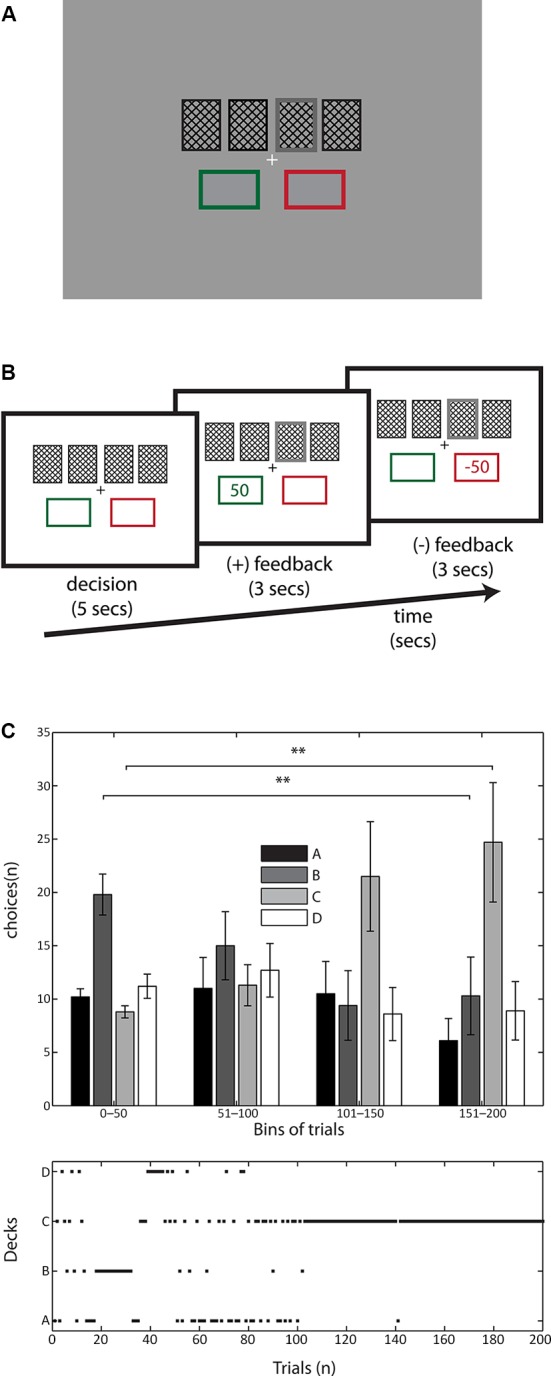
**Display depiction, trial timeline and behavioral results in IGT. (A)** Schematic of the display. The colors of the stimuli were isoluminant to prevent pupil changes due to luminance, and the relevant stimulation was kept near the center of fixation in order to prevent saccades. The four decks were represented by cards presented 1° above the fixation cross. After a selection the card was highlighted (double line stroke and 80% gray, here depicted in deck 3). The two rectangles holding the positive (green outline) and negative (red outline) were kept during the whole trial.**(B)** A typical trial started with a fixation cross followed by the decision screen where the participants were asked to pick one of the four decks (5 s), immediately after the selection the chosen deck was highlighted and the PF was presented (3 s) followed for the NF (3 s). **(C)** *Top:* Temporal evolution of subjects’ choices across the experiment, trials were pooled in 50 trials’ bins. Note the interaction between time (trials) and deck selection especially in the rising and sinking in preferences of decks **C** and **B**, respectively. Data depicted as mean +/− SEM. *Bottom:* Example of trial-by-trial evolution of deck selections of one typical subject. Note the progressive change in preferences towards deck **C**.

The task was modified in order to equalize the luminance of the display for the pupillometry and to stabilize the amount of winnings and punishment of the task. Equalized luminance was achieved by tuning each color used in the display to yield ~15 cd/m^2^ (see Figure 1A). The stimulation was presented in a 19” CRT monitor located at 70 cm from the participants. We recorded participants’ pupil diameter at a sample rate of 1 kHz with an infrared eye-tracker system (EyeLink 1000, SR Research^1^), in between each series of trials the eyetracker was calibrated with a nine point standard grid provided by the manufacturer. Data analysis was performed with custom routines written in MatLab (The MathWorks, Inc., Natick, MA): First, raw signals were low-pass filtered with a Savitzky-Golay^2^ filter (Savitzky and Golay, 1964) (polynomial degree: 2, span: 10). Second, pupil signal was averaged across both eyes and finally locked to the onset of feedback. When the signal was zero in both eyes it was interpreted as a blink period and the pupil dilation signal was inferred through cubic spline interpolation. Subjects were instructed to blink before feedback presentation in order to minimize blinking periods.

### Experimental Procedure

Like in the original IGT, subjects started with a virtual loan of $2000. They were told that the game consisted in a long set of card selections, one card at a time. Subjects were asked to pick one card from four virtual decks (A, B, C and D) by pressing the numbers 1, 2, 3 or 4 respectively in a standard computer keyboard. They were instructed that cards from each decks always involved a standard winning (following the selection and varying with the decks) and that sometimes they may receive a punishment (appearing after the positive feedback (PF), and varying with the decks as well) altogether with the winning. Participants first faced the decision screen in which they had 5 s for picking a card from one of the four decks. If they did not choose any card then the trial was repeated. After the selection of the card, the PF was presented for 3 s occasionally followed by a NF presentation for 3 s either (see Figure 1B). In the cases where no NF was presented the next trial started after the PF. Participants played two series of 100 trials each with a 5 min break between them. The instructions were preserved from the original IGT, where participants were told that the goal of the game was to maximize their winnings, and they were free to switch from any deck to another at any time. Subjects were neither told about the probability and magnitude distribution over the decks nor how many trials they had to play. They were told that there was a base payment of €15 for their participation, followed by a bonus based on their performance. However, all the subjects were equally rewarded with €25 regardless of their performance at the end of the experiment. This information was provided to the subjects after the experiment.

### Experimental task

The underlying distribution of rewards and punishments was maintained from the original IGT as well as the order in which the cards were presented through each deck (Bechara et al., 1994), with small changes in the quantities of the feedbacks. Decks A and B always presented a winning of $100, and while deck A had a punishment of $-250 with 0.5 of probability, deck B had a $-1250 punishment with 0.1 of probability. Both decks had a net value (NV) of $-250 per 10 cards. The change regarding the traditional IGT is that the punishment of the decks A was standardized to $-250, while in the original it fluctuated between $-150 and $-350. This change did not affect the NV, which remained the same. This was done in order to exclude possible effects produced by the novelty of having different feedback magnitudes in each trial of the same deck. Decks C and D always presented a winning of $50, and while deck C had a punishment of $-50 with 0.5 of probability, deck D punished with $-250 with 0.1 of probability. Both decks had a NV of $250 per 10 cards, being the most convenient options for the participants. Deck C was changed in the same way than deck A and for identical reasons, but again the NV was maintained as in the original IGT (Bechara et al., 1994).

## Results

### Behavioral results

In order to assess subjects’ behavior in the IGT, we analyzed the behavioral data with a two-way ANOVA, with *time (blocks)* and *deck* as factors. We found a main effect of *deck* (*F*(3, 144) = 4.73, *p* < 0.05) as well as a significant *interaction* between *time* and *deck* (*F*(9, 144) = 3.4, *p* < 0.001). This shows that the time on which participants interacted with the task shaped their decisions. The IGT structure comprises two convenient decks (C and D, NV = 250) and two inconvenient ones (A and B, NV = −250), therefore task learning is expressed when subjects progressively prefer the convenient decks over the inconvenient ones throughout the time of the task (Bechara et al., 1994; Figure 1C).

To explore the effect of learning we pooled the data into four bins of 50 trials each, thus we could observe the distribution of deck selections throughout the task. Results show that participants started preferring deck B (see first bin in Figure 1C), but over the time their selection moved toward deck C (see last bin in Figure 1C) suggesting that subjects indeed pursued a strategy to maximize their benefits from the task. We ran a post-hoc paired *t*-test comparing the frequency of elections of deck B in the first and fourth bins, and the same comparison was performed for deck C. We found that deck B was chosen significantly more by subjects in the first 50 trials than in the last 50 selections (paired *t*-test (9) = 3.1540, *p* < 0.01). We found an inverse relation with deck C, which was significantly more chosen by participants toward the end of the task compared to the first bin (paired *t*-test (9) = −2.9198, *p* < 0.01; see Figure 1C).

### Pupillometry

Pupil diameter data was analyzed in two periods of interest, *positive* and *negative* feedback periods associated with uncertainty and surprise coding respectively. For analysis of PF and NF, pupil traces were aligned to the time of both feedback presentations. All traces were normalized by subtracting and dividing by the diameter at the time point of the feedback presentation, thus we obtained a percentage signal chance measure relative to PF and NF. This normalization prevents confounds derived from effects that last longer than individual trials (Preuschoff et al., 2011).

During PF period the uncertainty is related to the unknown probability associated with receiving a future NF when choosing a card. Thus, when this probability is 0.5 there is higher uncertainty about an actual result (decks A and C), than when the probability is 0.1 (decks B and D). During PF, surprise cannot be examined since in the instructions subjects are told that they will always receive a standard PF. During NF period surprise is defined as a function of probability (Qiyuan et al., 1985; Preuschoff et al., 2011). Following, Preuschoff et al. (2011), NFs from decks with low probability (decks B and D) are expected to elicit greater surprise than NFs with high probability (decks A and C) since NFs with lower probability generate expectations that are not met when the feedback appears. The normalization of the pupil trace explained above was conducted in order to eliminate interferences of PF’s responses over NF period.

**Figure 2:**
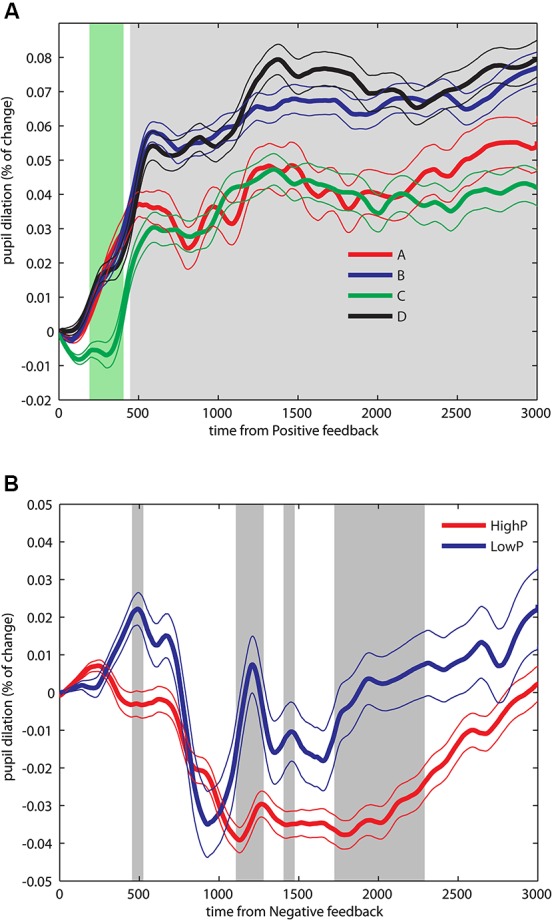
**Pupil dilation dynamics during an IGT. (A)** Pupil dilation dynamics during PF. An ANOVA performed over successive data points over the whole PF period, yielded a main effect for NF magnitude during a brief period after positive reward presentation (200–400 ms, green shaded area) as well as a main effect for NF probability for a sustained period starting at 500 ms until the end of the period (gray shaded area). **(B)** Pupil dilation dynamics during NF. Data from four decks were pooled by NF probability in *HighP* decks (A and C), and *LowP* decks (B and D). Significant differences were found during a brief period around 500 ms, and in two later sustained periods ranging from 1200–1300 and 1700–2400 ms. Significant results are reported after FDR correction, *p*_FDR_ < 0.05. Data depicted as Mean +/− SEM.

First, we explored the PF period, where uncertainty-related pupil modulations were expected. To control family-wise error rate, *p*-values were corrected for multiple comparisons with the *false discovery rate* (FDR) method of Benjamini and Hochberg (1995). We analyzed the data distributions with an ANOVA with PF* magnitude*, *NF probability* and *NF magnitude* as factors. Because we found neither main effects nor interactions involving *PF magnitude* (*p* > 0.05) we moved to a more parsimonious model including two factors (*NF probability* and *NF magnitude*) and its potential interactions. We found a main effect of *NF magnitude* for a brief period after positive reward presentation (200–400 ms, *p*_FDR_ < 0.05) followed by a main effect for *NF probability* for a sustained period beginning at 480 ms until the end of the positive reward window (*p*_FDR_ < 0.05, see Figure 2A). The early effect of NF magnitude was present just during a brief early period and may be due to a decision-related response more than a feedback-evoked effect. Thus, in the PF period, an anticipatory response was consistently found in that greater pupil dilation change appears when a future NF was unlikely (decks B and D), compared to uncertainty about future NF (A and C). Interestingly, there were no sustained differences between decks B and D, and neither between decks A and C, showing that the magnitude of NF presentation was not relevant.

**Figure 3:**
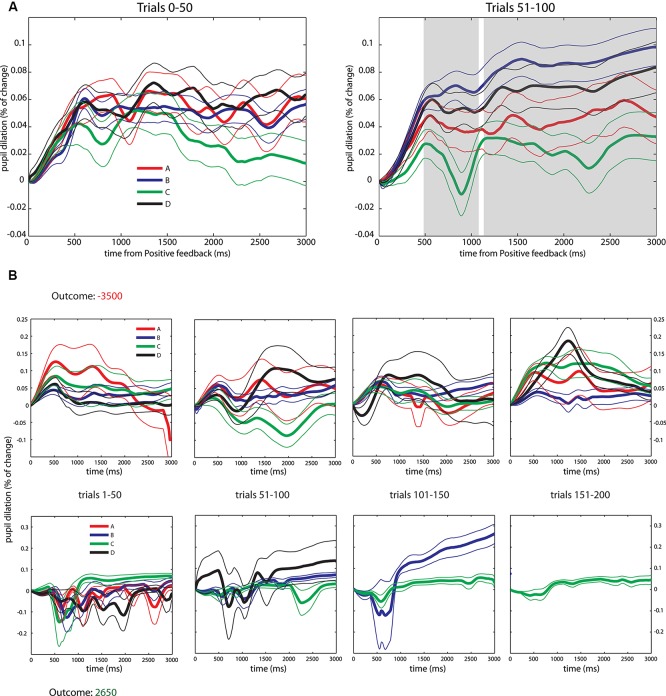
**Pupil dilation dynamics during PF in the first half of the IGT. (A)** Average across all subjects. *Left*: Pupil responses to PF in the first 50 trials, note the overlapping of traces. *Right*: Pupil responses to PF in the trials 51–100, note the formation of the general pattern reported in the pooled data and statistical differences arising in similar periods (500 ms to the end of the period, gray shaded area). ANOVA and FDR correction identical as the applied to pooled data. Significant results are reported after FDR correction, *p*_FDR_ < 0.05. **(B)** Pupil dilation dynamics during PF across the whole duration of the IGT for two subjects with extreme performances, data organized in four bins of 50 trials. *Top*: Subject with worst outcome. *Bottom*: Subject with best outcome. All data depicted as Mean +/− SEM.

Following this, and in order to measure learning responses over the task, we pooled the data of PF into four bins of 50 trials each and compared the pupil dilation changes over the first and second bins. Observing behavioral data (Figure 1C) it is possible to see that while the first bin (from trials 1–50) is mainly exploratory, in the second block (trials 51–100) trends of selection start to appear despite that there is still dispersion over the deck selections. On the other hand, in bins 3 and 4 (from trials 101–200) the deck selection is much more concentrated in deck C, which entails that there are not enough trials from each deck choice for conducting a statistical comparison (see Figure 3B for a depiction of a good and poor performer in the task). Thus, when analyzing pupil dilation over the first 50 trials we found no pupil differences between the four decks (*p*_FDR_ > 0.05, see Figure 3A). However, when analyzing pupil dilation responses in the second bin we did find the effects previously exposed. We found greater pupil dilation changes in decks B and D, compared to decks A and C (*p*_FDR_ < 0.05) beginning at 500 ms and sustained through the rest of the feedback period (with a brief interruption around 1100 ms, see Figure 3A) emulating the effect in uncertainty modulation found in the grand averages.

Next, we examined the NF period, where we predicted surprise related pupil responses. Following the main effect of *feedback probability* in the PF period, the null effect in *PF magnitude* and the fewer number of trials including a NF, we pooled the trials according to probability of negative outcomes by high (*HighP*; decks A and C) and low (*LowP*; decks B and D) probability. Significant differences (*p*_FDR_ < 0.05) were found in three periods, an initial brief period around 500 ms, and in two later periods ranging from 1200–1300 and 1700–2400 ms (see Figure 2B). Thus, NF with lower probability elicited greater pupil dilation changes (decks B and D), compared to NF with higher probability (decks A and C). Following the same procedure as described for PF, we pooled the data into four bins and compared the first two bins with the aim to assess differences in pupil responses over the duration of the experiment. No differences were found in the first bin, and only brief periods of significant differences were found in the second bin (100–200 and 600–750 ms, data not shown).

## Discussion

The results demonstrate that subjects learned to increase their winnings during the game and exhibit a consistent effect of uncertainty and surprise on pupil dilation. In our paradigm, uncertainty is related to the known probability of getting feedback. Given the structure of the IGT we interpreted PF presentation as the uncertainty condition, and NF presentation as the surprise condition. The IGT feedback presentation always involved PF first (probability = 1), and then NF whose probability was unknown to participants. The distribution of NF probability over the four options had two decks with 0.5 probability (decks A and C) defined as high uncertainty, and two decks with 0.1 probability (decks B and D) defined as low uncertainty. The division of uncertainty conditions based upon risk has been traditionally used as a consistent model of experimental decision-making tasks (Weber et al., 2004; Mohr et al., 2010; Preuschoff et al., 2011). Surprise is defined as an improbable result over an expectable scenario. This means that in low uncertainty NF decks are more surprising compared to high uncertainty decks.

In the IGT, subjects do not have any knowledge of the probability and magnitude distributions of the NF in the four decks, besides their own experience after receiving the feedback. In this sense, our paradigm differs from previous pupil dilation studies (see Satterthwaite et al., 2007; Preuschoff et al., 2011) in that the risk involved in the participants’ choice is learned. The probability and magnitude distributions of the game contain two inconvenient decks (A and B), and two convenient ones (C and D). Thus, subjects can learn based upon their experience to avoid the inconvenient decks in order to increase their winnings. Behavioral results indicate that subjects learned to avoid inconvenient decks (A and B) in favor of at least one of the convenient ones (deck C, see Figure 1C). This finding is consistent with the trend found in the original study using the IGT (Bechara et al., 1994), and indicates that subjects were engaged in the task.

The uncertainty effect is found during PF presentation, where the certainty level about future NF modulated subjects’ pupil dilation from 480 ms until the end of the PF epoch. First we conducted an analysis over the whole deck selection distribution in which we found greater pupil dilation changes in low uncertainty decks (B and D) without differences between them, compared to the high uncertainty ones (A and C) which also did not present differences between them. Complementary to this analysis, we conducted an analysis comparing pupil responses from the first 50 trials to the second 50. We ran this analysis in order to see whether pupil responses were actually modulated differently once subjects learned how to perform the task. We found no differences in pupil responses during the first time block (trials from 1–50), but we did find differences in the second block (trials 51–100) that show the trend of the results observed out of the total deck selection. This suggests that pupil responses signal learned uncertainty of the different probability conditions of decks A–C and B–D. Given the structure of the task (in that the number of trials per deck depends on subjects’ selections), the final 100 trials are highly concentrated over deck C, which makes a comparison of pupil responses difficult due to the lack of trials for the rest of the decks. Moreover, in Figure 3B we included a graphical comparison of the subject who got the worst payoff in the IGT to the one that obtained the best payoff. This case illustrates the concentration of decisions found in good players and the dispersion found in the poor performers.

This consistent effect suggests that pupil dilation works as a learning marker of the different conditions of certainty during a decision-making task. These results replicate the findings of Preuschoff et al. (2011) and add information about the responses of pupil dilation facing uncertainty in learning conditions. In the Preuschoff et al. (2011) paradigm, subjects had to bet whether one card had higher value than another one in a scale from 1–10. Thus, after knowing that the first card was 1 there was absolute certainty that the second was higher, but when the first card was 5 there was high uncertainty about the future outcome. In this paradigm subjects start the game knowing those conditions of certainty/uncertainty. However, in the IGT this is not the case and subjects also have to learn the different conditions of the game in order to improve their performance. Our results suggest that pupil dilation changes serve as marker of not only responses facing different certainty levels, but also as a marker of learned uncertainty about future feedback conditions using bottom-up information.

The observation of pupillary responses toward the uncertainty conditions of the IGT also contributes to the understanding of cognitive mechanisms involved in the successful performance of decision-making under uncertainty. The somatic marker hypothesis (Bechara et al., 1994; Martinez-Selva et al., 2006) has been proposed to explain the physiological mechanisms responsible for successful performance in decision-making scenarios under uncertainty, as in the IGT. This hypothesis points out that in successful IGT performers, somatic responses differentiate NF and PF and that such responses work afterwards as anticipatory markers of risky decisions (Bechara et al., 1994; Martinez-Selva et al., 2006). Crone et al. (2004) using an adapted version of the IGT, reported greater heart rate slowing and skin conductance levels in subjects before choosing disadvantageous options compared to advantageous ones, but only in good performers. These responses were reported as critical to explain the physiological differences between bad and good performers in the IGT. Our results contribute to understanding processes that may work in parallel to such anticipatory responses, related to the signaling of uncertainty levels involved in learning tasks. While the anticipatory responses reported by Crone et al. (2004) were sensitive to feedback magnitudes, pupil dilation is sensitive to the level of uncertainty comprised in the different decks regardless of the magnitude of the feedback. This goes in the direction of a hypothesized risk-prediction error system that is thought to be involved in the identification of levels of uncertainty (Preuschoff et al., 2011). An interesting future study could replicate the Crone et al. (2004) experimental design and include investigating pupillary responses of different groups of players in order to evaluate whether pupillary sensitivity to uncertainty levels is impaired in bad performers. This could provide further evidence for the NA involvement in this risk-prediction error system (D’Acremont et al., 2009; Preuschoff et al., 2011).

Results also show a trend in pupil dilation responses related to surprise. As we stated before, in the IGT surprise appears during NF presentation, since subjects at this point do not experience more uncertainty (they are informed about the final outcome of their decision). Results from the two high uncertainty decks (A and C) are less surprising since there are no specific expectations about them (their probability of appearance is 0.5). On the other hand, when subjects can be more certain about the outcomes (decks B and D) higher surprise is predictable if their expectations are not met (Preuschoff et al., 2011). This implies that when subjects are expecting not to have a NF, surprise is expected when they actually receive such feedback. Results show that there were greater pupil dilation changes during surprising NF presentation, compared to low surprising decks. This result is consistent with evidence relating improbable or rare stimuli to pupil dilation (Brown et al., 1999; Siegle et al., 2008; Raisig et al., 2010). However, in our study surprise is not merely related to the oddity of a given stimuli, but to the exploratory behavior of subjects while learning under risk.

In order to measure the different pupillary responses during the task, we compared pupillary reactions between the first two bins of 50 trials (trials 1–50 and 51–100 respectively). We did find differences in pupil dilation, but this effect was not sustained throughout the whole interval of feedback presentation. We believe that this shows a trend in that the knowledge about the task modulated, though not steadily, pupil responses indicating differential processing of improbable feedback. This effect, although not conclusive, matches with evidence suggesting that pupil dilation signals errors related to uncertainty estimations, being a marker of surprise when a given feedback does not meet expectation (Preuschoff et al., 2011). This interpretation also matches with the relation between NA and unexpected uncertainty signaling (Yu and Dayan, 2005). Although the evidence is indirect, it goes in the direction that greater pupil dilation related to surprise can be taken as a marker of NA signaling errors to judge uncertainty, process that ultimately enhances task learning. Thus, since the violation of expectations involves a constant updating of the mental representations about underlying probability distributions, surprise might play a relevant role in suppressing top-down expectations in order to adapt behavior to a convenient strategy (Yu and Dayan, 2005). Further research is needed in order to provide better support for this interpretation, and overcome the issue of the fewer numbers of trials that IGT offers of NF presentation. Since the IGT is a learning task, toward the end of the game the distribution of election is progressively concentered on one deck. There is also the issue of task relevant stimuli that may be affecting pupil responses independently of the processing of an informative feedback.

Our paradigm measured pupil dilation in a decision-making task that involves active learning from subjects in order to adapt their behavior towards a convenient strategy. Results indicate that subjects learned how to face the task in order to increase their winnings. Pupil dilation was modulated by the different levels of uncertainty that the decks offered to participants, which had the features of an anticipatory response in the experiment. Although PF presentation was chronologically in first place, it was during this epoch that subjects exhibited pupil dilation modulation related to the learned risk of a future NF. After NF presentation, subjects’ pupil dilation was modulated by the level of surprise of the outcome, signaling estimation errors that potentially contribute to the task learning process. These results suggest that pupil dilation serves as a marker of learned uncertainty and support previous evidence that demonstrates the association of pupil dilation to surprise. These results also provide indirect support for the hypothesis that pupil dilation may serve as a marker for NA changes modulated by external cues that help people adapt their behavior within unfamiliar contexts.

## Conflict of interest statement

The authors declare that the research was conducted in the absence of any commercial or financial relationships that could be construed as a potential conflict of interest.
